# Ankyloblepharon filiforme adnatum: a case report

**DOI:** 10.11604/pamj.2013.15.15.2209

**Published:** 2013-05-08

**Authors:** Bouchra Alami, Asmae Maadane, Rachid Sekhsoukh

**Affiliations:** 1 Ophthalmology Department, El Farabi Hospital, Medical School, University Mohammed First, Oujda, Morocco

**Keywords:** Ankyloblepharon filiforme adnatum, malformation, eyes

## Abstract

Ankyloblepharon is defined by partial or complete adhesion of the ciliary edges of superior and inferior eyelids. It is usually a sporadic isolated malformation in which the upper and lower lids are joined by tags. Although it is an uncommon and benign condition, its presence should alert the clinician to the possibility of other important disorders. We report a case of a new born who had a sporadic ankyloblepharon, treated one day after birth.

## Introduction

Ankyloblepharon describes direct fusion of the lids. It is a rare congenital abnormality, in which single or multiple strands of fine connective tissue join the upper and lower lids anywhere along the lid but never at the lateral or medial canthus [1]. These strands are extensible, and by forcibly opening the lids their length can be almost doubled. The tissue invariably arises from the grey line, anterior to the meibomian gland orifices and posterior the cilia.

## Patient and observation

**Figure 1:**
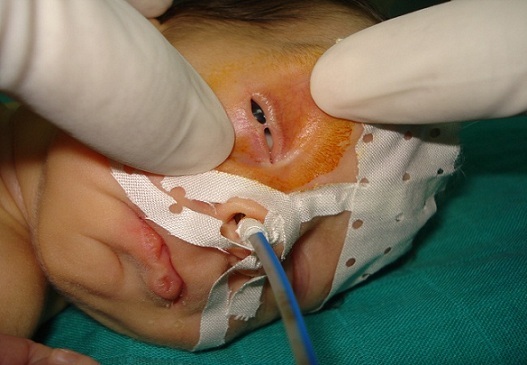
Photograph of the neonate with ankyloblepharon filiforme adnatum showing partial fusion of the right upper and lower eyelids by a band of tissue

**Figure 2:**
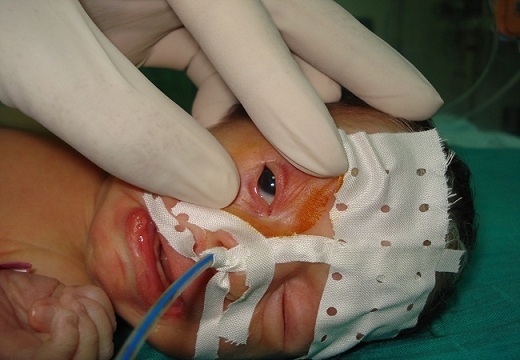
Right eye immediately after adhesion separation

A female neonate was referred for assessment of his right eyelids. She was born at term, to a primigravid mother, weighing 3400 g. Pregnancy and delivery were unremarkable. There was no family history of congenital anomalies or consanguinity. The baby was noted to have fused right eyelids at birth; apart from this, she was perfectly healthy. A detailed pediatric assessment failed to reveal any other congenital abnormalities. Ocular examination showed partial fusion of his right upper and lower eyelids by a central narrow band of tissue, arising from the grey lines (Figure 1). Full eyelid opening was impaired and interpalpebral aperture was limited to 3.5 mm. Right eye examination was normal. The band of tissue was excised with Vannas scissors at the level of each eyelid margin (Figure 2). The procedure was performed under sedation. There was a limited bleeding. Subsequent right eye examination did not reveal any other pathology. At her follow up appointment 5 months later, no abnormality was noted.

## Discussion

Ankyloblepharon filiforme adnatum (AFA) is a rare benign congenital anomaly, first described by Von Hasner in 1881. Fusion of the eyelid margins is a normal stage in human development, but an abnormal occurrence at birth. The developing eyelid margins remain fused until the fifth gestational month, but may not be completely separated until the seventh month. Ankyloblepharon is characterized by full thickness fusion of the lid margins.

AFA describes single or multiple bands of tissue joining the upper and lower eyelids either unilaterally or bilaterally. The histology of these connecting strands has been shown to consist of vascularised central core surrounded by stratifed squamous epithelium [1]. It may present as an isolated congenital defect such as in our patient. However, it is always important to actively look for coexisting pathology.

The ophthalmic association of AFA is iridogoniodysgenesis with juvenile glaucoma [2]. It has been reported in the context of trisomy 18 (Edwards′ syndrome) [3], Hay-Wells syndrome (a variant of the ectodactyly-ectodermal dysplasia-cleft lip palate syndrome) [4, 5], the popliteal pterygium syndrome (characterized by intercrural webbing of the lower limbs), and CHANDS (curly hair-ankyloblepharon-nail dysplasia syndrome). Other associations may include hydrocephalus, meningocele, an imperforate anus, bilateral syndactyly, infantile glaucoma and cardiac problems such as patent ductus arteriosus and ventricular septal defects [6].

Detailed systemic assessment by an experienced pediatrician is therefore imperative in the management of AFA.

Rosenman et al [7, 8] divide AFA into four subgroups (Table 1) and indicated that groups I and II were sporadic and groups III and IV were autosomal dominant with variable expressivity. Bacal et al [3] suggested a fifth group: AFA in association with chromosomal abnormalities.

**Table 1:** Classification of ankyloblepharon filiforme adnatum

Group	Associated abnormalities
**I**	None
**II**	Cardiac or central nervous system
**III**	Ectodermal syndrome
**IV**	Cleft lip and/or palate

The aetiology of this abnormality is unknown and a number of theories have been proposed. The currently accepted theory is that this condition is due to an interplay of temporary epithelial arrest and rapid mesenchymal proliferation, allowing union of lids at abnormal positions [5].

This case report demonstrates the simplicity in treating AFA. Surgical correction should be performed promptly to minimize any risk of amblyopia, and enable full examination of the eye. It also highlights that its presence should alert the clinician to the possibility of an underlying congenital disorder.

## Conclusion

Ankyloblepharon filiforme adnatum is a rare but potentially amblyogenic congenital abnormality of the eyelids. Treatment should be performed to minimize the risk of amblyopia.
